# Retraction Note: Novel Therapeutic Approach Using Drug-loaded Adipose-derived Stem Cells for Pancreatic Cancer

**DOI:** 10.1038/s41598-021-99148-5

**Published:** 2021-09-28

**Authors:** Masahiko Aoki, Kazuki Kakimoto, Masahiro Goto, Kazuhide Higuchi

**Affiliations:** Internal Medicine (II), Faculty of Medicine, Osaka Medical College, Osaka, Japan

Retraction of: *Scientific Reports* 10.1038/s41598-019-53807-w, published online 29 November 2019

The Authors have retracted this article because they are no longer confident that all of the adipose tissue used in this study was obtained under the protocol approved by the Ethics Committee.

All Authors agree with the retraction and its wording.

